# Social, behavioral, and sleep characteristics associated with depression symptoms among undergraduate students at a women’s college: a cross-sectional depression survey, 2012

**DOI:** 10.1186/1472-6874-14-8

**Published:** 2014-01-13

**Authors:** Katherine T Wilson, Ashley E Bohnert, Alex Ambrose, Destiny Y Davis, Dina M Jones, Matthew J Magee

**Affiliations:** 1 Agnes Scott College Department of Public Health, 141 East College Avenue, Decatur, USA; 2 University of Minnesota School of Public Health, Division of Epidemiology & Community Health, 420 Delaware Street Southeast, Minneapolis, USA; 3 Emory University Rollins School of Public Health, Department of Epidemiology, 1518 Clifton Road Northeast, Atlanta, USA

**Keywords:** Mental health, Depression, Sleep, Women’s college

## Abstract

**Background:**

The association between student characteristics and depression among students attending women’s colleges (single-sex institutions of higher education that exclude or limit males from admission) is poorly understood. Our objective was to estimate the prevalence of depression and determine behavioral and social characteristics associated with depression among students attending a women’s college.

**Methods:**

We administered a cross-sectional Internet-based survey between April and May 2012 to students (n = 277) enrolled at a private women’s college in the southeastern US. Center for Epidemiologic Studies Depression (CES-D) and Depression Anxiety Stress Scale 21 (DASS-21) instruments measured self-reported depression. Bivariate and multivariable logistic regression methods were used to estimate adjusted associations.

**Results:**

Prevalence of depression measured by CES-D and DASS-21 instruments was 26.3% (95% confidence interval [CI] 20.8-32.3%) and 26.0% (95% CI 20.4-32.3%), respectively. After adjusting for confounders, absence of strong social support (prevalence odds ratio [OR] = 4.3, 95% CI 1.4-13.7), history of mental health disorder (OR = 4.8 95% CI 1.9-12.4), and poor sleep hygiene (OR = 2.8, 95% CI 1.3-5.8) were associated with depression.

**Conclusions:**

This cross-sectional survey identified absence of strong social support, history of mental health disorder, and poor sleep hygiene as potential predictors of depression among students attending a women’s college. Further investigation of these factors may inform depression interventions for students attending women’s colleges and other undergraduate student populations.

## Background

Depression is a frequently reported mental disorder in the US college student population. In the spring 2012 American College Health Association National College Health Assessment of students from 141 self-selecting postsecondary education institutions, over one-fifth of undergraduate students reported a current or previous diagnosis with depression [1]. Studies have identified depression among college students as a risk factor for poor academic performance [2,3], problem drinking [4], suicidal thoughts [5,6], frequent illness [7], and college dropout [8].

Several risk factors for depression among US college students have been investigated, but debate remains regarding which factors are consistently associated with increased risk of depression. Studies have identified lack of certain types of exercise [9-11] and sleep quantity [12] as likely risk factors. Poor sleep quality among college students is also predictive of poor academic performance [13,14], problem drinking [15], and suicidal thoughts [16]. Among adolescents, short sleep duration is associated with suicidality, illicit drug use, and depression defined by the Center for Epidemiologic Studies Depression (CES-D) scale [17]. Poor sleep quality and depression are likely to have a bidirectional relationship among young adults [18].

Studies among undergraduate students have reported non-significant differences between the prevalence of depression among female and male undergraduate students in the US [19-21]. However, in a national report that assessed the US prevalence of diagnosed or treated depression among college students, 12.5% of females versus 6.7% of males reported depression in the previous year (p-value <0.001) [1]. Prospective observational studies among female undergraduate students have identified sleep deficit [12], lack of certain types of exercise [22], and binge eating [23] as predictors of depression.

Whether national trends in depression and risk factors for depression are similarly distributed at women’s colleges (single-sex institutions of higher education that exclude or limit males from admission) remains under-investigated. Female students attending women’s colleges have been found to have greater academic involvement [24,25], greater self-confidence [25,26], and higher satisfaction with college experience [24,27] but lower satisfaction with quality of social life/interpersonal support [27,28] compared to female students attending co-educational institutions. Differences in satisfaction with educational experiences at women’s colleges, compared to co-educational colleges, may also affect prevalence of depression. However, few studies have assessed depression symptoms, student characteristics, or the relationship between student characteristics and depression at women’s colleges [12]. Given the paucity of data on depression among women’s colleges in the US, we aimed to 1) estimate the prevalence of depression symptoms among female undergraduate students at a women’s college and 2) determine behavioral, social, and sleep characteristics associated with depressive symptoms among the undergraduate students.

## Methods

### Design and data collection

A cross-sectional survey was implemented at a private women’s liberal arts college in the southeastern US. All current students, enrolled during the 2011 to 2012 academic year, were eligible to participate in the study. A 47-question, computer-based survey was administered via a commercially available Internet application between April and May 2012. All current students received three recruitment e-mails; those who consented were linked to the Internet survey. The survey could be completed from any computer with Internet connection. Participation was voluntary, and responses were anonymous.

### Ethics statement

The Agnes Scott College Institutional Review Board approved the study protocol and questionnaire. Informed consent was obtained from all participants via the Internet survey tool.

### Measures

The survey instrument collected self-reported information on students’ demographics, individual sleep behaviors, perceptions of mental health disorders, and social network characteristics. Self-reported measures were drawn from the US Center for Disease Control’s 2012 Behavioral Risk Factor Surveillance System Questionnaire [29], or were collected using single-item questions designed by the researchers. The survey also included the Center for Epidemiologic Studies Depression (CES-D) 20-item version to assess frequency of recent depressive symptoms [30]. A CES-D score was calculated for each student as previously described [30]. Current CES-D depression was defined as a score ≥ 16 [30]. In the same Internet survey, the short form of the Depression Anxiety Stress Scale (DASS-21) 21-question assessment was also administered [31]. Current DASS-21 depression was defined as a score ≥ 14 as previously described [31].

### Statistical analysis

Data collected via the Internet survey tool were automatically stored in an electronic database. All statistical analyses were performed using SAS Version 9.3 (SAS Institute Inc., Cary, NC). Bivariate analyses were used to determine the association between individual characteristics and current CES-D and DASS-21 depression. For bivariate analyses, the Cochran-Mantel-Haenszel chi-square test was used to calculate p-values for categorical variables, and the Student’s t-test (for normally distributed) or Wilcoxon–Mann–Whitney rank sums test (for non-normally distributed) was used for continuous variables. A two-sided p-value < 0.05 was considered statistically significant throughout the analyses. Student characteristics associated with current depressive symptoms (defined by CES-D and DASS-21) with a p-value < 0.2 were entered in predictive binomial multivariable logistic regression models using backward and stepwise elimination with a p-value < 0.05 required for retention. A multivariable logistic causal (non-elimination procedure) model was also used to estimate the adjusted association between sleep quality and current depressive symptoms. Confounders included in the causal model were chosen based on observed associations in the collected data, directed acyclic graph theory [32], and previous literature.

## Results

During the spring semester of 2012, 305 of 820 (37.2%) students consented to the survey. Among students who consented, 22 (7.2%) were removed because they did not begin the survey, 6 (2.0%) were ineligible because they were not enrolled in the undergraduate program, and 277 (90.8%) were included in the analyses. Among the included participants, 240 (85.7%) completed the CES-D and 227 (81.1%) completed the DASS-21 portions of the survey instrument.

Most participants were residential students (80.7%), U.S.-born (87.1%), and non-Hispanic African American/Black (29.1%) or non-Hispanic White (51.5%). The mean and median ages were 21.4 (standard deviation [SD] 5.0) and 20.4 years (interquartile range [IQR] 2.2), respectively. Class representation was approximately equal, with at least one-fifth of participants representing each class. The majority of participants (96.1%) were enrolled as full-time students, taking at least 12 credit hours.

Among 240 participants who completed the CES-D portion of the survey instrument, 63 (26.3%, 95% CI 20.8—32.3%) had a score ≥ 16, indicating current depression (Table 1). The mean and median CES-D scores were 12.4 (SD 9.8) and 10 (IQR 10), respectively. Of 227 participants who completed the DASS-21 portion of the survey instrument, 59 (26.0%, 95% CI 20.4—32.3%) had a score ≥ 14, indicating current depression. Participants had a mean DASS-21 score of 9.5 (SD 9.6) and median DASS-21 score of 6 (IQR 12). Depression by CES-D and DASS-21 had 83.6% agreement (Kappa value 0.57, p-value <0.001).

**Table 1:** Distribution of CES-D/DASS-21 depression among students attending a women’s college, 2012

**Mental Health Scale**	**Univariate statistic**
CES-D score (N = 240)	
Mean (SD)	12.4 (9.8)
Median (IQR)	10 (10)
Range	54
Percent ≥16 score (Exact 95% CI)^a^	26.3% (20.8-32.3%)
DASS-21 score (N = 227)	
Mean (SD)	9.5 (9.6)
Median (IQR)	6 (12)
Range	42
Percent ≥14 score (Exact 95% CI)^b^	26.0% (20.4-32.3%)
Percent agreement	83.6%
Kappa statistic^c^	0.57, p-value <0.001

*Note.*^a^CES-D score ≥16 is defined as moderate depressed mood by Radloff [30].

^b^DASS-21 score ≥14 is defined as moderate to extremely severe depression by Lovibond and Lovibond [31].

^c^Assessed agreement between moderately depressed mood by CES-D and DASS-21.

Self-reported overall health, family history of depressive and/or anxiety disorders, and history of clinical diagnosis or treatment for a mental health disorder were significantly associated with current depression by both CES-D and DASS-21 scales (Table 2). Self-reported body mass index (BMI) and average weeknight hours of sleep were significantly associated with CES-D and DASS-21 depression by the Wilcoxon–Mann–Whitney test. Both the self-reported number of social support group members and absence of strong social support group were significantly associated with depression by both scales.

**Table 2:** Association between CES-D/DASS-21 depression with individual behavioral, social, and community characteristics

	**Measure of depression**			
	**CES-D Score**		**DASS-21 Score**	
**Participant Characteristic**	**≥ 16**^**a**^	**<16**^**a**^	**≥ 14**^**a**^	**< 14**^**a**^
	**N (%)**	**N (%)**	**N (%)**	**N (%)**
	**63 (26.3)**	**177 (73.8)**	**59 (26.0)**	**168 (74.0)**
Age				
Mean (STD)	21.4 (5.0)	20.9 (2.7)	21.5 (5.2)	20.9 (2.7)
Median (IQR)	20.3 (2.2)	20.4 (1.8)	20.2 (2.4)	20.4 (1.8)
17–18	4 (6.7)	13 (8.0)	4 (7.2)	9 (5.8)
19–20	34 (56.7)	92 (56.8)	32 (58.2)	87 (56.5)
21–22	17 (28.3)	49 (30.3)	12 (21.8)	52 (33.8)
>22	5 (8.3)	8 (4.9)	7 (12.7)	6 (3.9)
Class year^c,d^				
Freshman	22 (34.9)	40 (22.7)	18 (30.5)	32 (19.5)
Sophomore	15 (23.8)	55 (31.3)	16 (27.1)	52 (31.7)
Junior	12 (19.0)	51 (29.0)	11(18.6)	50 (30.5)
Senior	14 (22.2)	30 (17.0)	14 (23.7)	30 (18.3)
Race/ethnicity				
African American/Black (non-Hispanic)	15 (25.0)	51 (29.7)	13 (23.6)	49 (29.9)
Asian	5 (8.3)	20 (11.6)	34 (61.8)	81 (49.4)
Hispanic/Latina	3 (5.0)	7 (4.1)	3 (5.5)	7 (4.3)
White (non-Hispanic)	35 (58.3)	85 (49.4)	2 (3.6)	19 (11.6)
Multiracial/ethnic	2 (3.3)	9 (5.2)	3 (5.5)	8 (4.9)
Nation of birth^c,d^				
United States	60 (95.2)*	150 (85.2)	56 (94.9)	144 (86.2)
Other	3 (4.8)	26 (14.8)	3 (5.1)	23 (13.8)
Residence^c^				
On-campus	47 (74.6)	148 (83.6)	46 (78.0)	138 (82.1)
Off-campus	16 (25.4)	29 (16.4)	13 (22.0)	30 (17.9)
GPA				
3.6-4.0	21 (33.9)	64 (36.6)	14 (24.1)	63 (37.7)
3.1-3.5	22 (35.5)	75 (42.9)	27 (46.6)	69 (41.3)
2.6-3.0	12 (19.4)	24 (13.7)	11 (19.0)	22 (13.2)
2.1-2.5	7 (11.3)	12 (6.9)	6 (10.3)	13 (7.8)
Number of credit hours^d^				
Mean (SD)	15.4 (4.0)	16.1 (3.0)	15.3 (4.1)	16.1 (3.0)
Median (IQR)	16.0 (2.0)	16.0 (2.0)	16.0 (2.0)	16.0 (2.0)
1–11	4 (6.8)	5 (3.0)	4 (6.8)	5 (3.0)
12–16	38 (64.4)	98 (58.7)	40 (67.8)	98 (58.3)
>16	17 (28.8)	64 (38.3)	15 (25.4)	65 (38.7)
Employment^d^				
On-campus	27 (42.9)	79 (44.9)	21 (35.6)	76 (45.5)
Off-campus	11 (17.5)	36 (20.5)	9 (15.3)	37 (22.2)
None	25 (39.7)	61 (35.7)	29 (49.2)	54 (32.3)
Self-reported overall health				
Excellent/very good/good^c,d^	47 (74.6)*	166 (94.9)	44 (77.2)*	157 (93.5)
Fair/poor	16 (25.4)	9 (5.1)	13 (22.8)	11 (6.5)
BMI^d^				
Mean (SD)	26.6 (7.8)*	24.1 (6.0)	27.1 (7.7)*	24.0 (5.9)
Median (IQR)	24.7 (8.4)	20.6 (6.1)	25.3 (9.2)	23.0 (6.0)
<18.5	5 (7.9)	13 (7.3)	3 (5.1)*	8 (4.9)
18.5-24.9	29 (46.0)	101 (57.1)	23 (39.0)	99 (60.7)
>24.9	29 (46.0)	63 (35.6)	33 (55.9)	56 (34.4)
Nightly hours of sleep^c,d^				
Mean (SD)	6.0 (1.6)*	6.7 (1.3)	6.0 (1.6)*	6.7 (1.3)
Median (IQR)	6.0 (2.0)	7.0 (1.0)	6.0 (2.6)	7.0 (1.0)
<5	12 (19.1)*	17 (9.6)	7 (11.9) *	5 (3.0)
5–6	32 (50.8)	67 (37.9)	32 (54.2)	69 (41.8)
>6	19 (30.2)	93 (52.5)	20 (33.9)	91 (55.2)
Sleep quality^c,d^				
Excellent/good/okay	35 (59.3)*	138 (82.6)	35 (59.3)*	140 (83.3)
Poor/extremely poor	24 (40.7)	29 (17.4)	24 (40.7)	28 (16.7)
Family member with previous mental health diagnosis^c,d^				
Depressive or anxiety disorder	9 (20.9)*	38 (27.9)	13 (30.2)*	34 (24.8)
Both	22 (51.2)	30 (22.1)	21 (48.8)	33 (24.1)
Neither	12 (27.9)	68 (50.0)	9 (20.9)	70 (51.1)
College loans^d^				
Yes	44 (69.8)	113 (63.8)	44 (74.6)	107 (63.7)
No	19 (30.2)	64 (36.2)	15 (25.4)	61 (36.3)
Major				
Undeclared	4 (7.0)	7 (4.5)	3 (5.2)	7 (4.5)
Humanities	11 (19.3)	20 (13.0)	10 (17.2)	22 (14.1)
Social sciences	21 (36.8)	66 (42.6)	24 (41.4)	63 (40.4)
Natural sciences	9 (15.8)	40 (25.8)	9 (15.5)	40 (25.6)
Double major	11 (19.3)	18 (11.6)	11 (19.0)	20 (12.8)
Dual-degree Nursing	1 (1.8)	4 (2.6)	1 (1.7)	4 (2.6)
Self-reported increase in stress from college				
Yes	53 (89.8)	138 (83.6)	53 (89.8)	142 (84.5)
No	6 (10.2)	27 (16.4)	6 (10.2)	26 (15.5)
Frequency of weekly academic stress^b^				
Often	44 (74.6)*	72 (43.1)	46 (78.0)*	70 (41.7)
Not very often	12 (20.3)	80 (47.9)	11(18.6)	82 (48.8)
Almost never	3 (5.1)	15 (9.0)	2 (3.4)	16 (9.5)
Number of people in social support group^c,d^				
Mean (SD)	5.1 (4.2)*	6.5 (5.3)	4.6 (4.0)*	6.7 (5.2)
Median (IQR)	4.0 (4.0)	5.0 (5.0)	3.5 (3.5)	5.0 (4.0)
0–4	30 (54.5)	62 (38.3)	35 (62.5)*	56 (34.6)
>4	25 (45.5)	100 (61.7)	21 (37.5)	106 (65.4)
Strong social support group^c,d^				
Yes	27 (58.7)*	128 (89.5)	25 (53.2)*	134 (92.4)
No	19 (41.3)	15 (10.5)	22 (46.8)	11 (7.6)
Relationship status^c^				
Single	42 (68.9)	96 (54.9)	34 (59.6)	92 (55.4)
Relationship	19 (31.1)	79 (45.1)	23 (40.4)	74 (44.6)
Children				
Yes	2 (3.2)	6 (3.4)	2 (3.4)	6 (3.6)
No	61 (96.8)	169 (96.6)	57(96.6)	162(96.4)
Exercise (≥ 1 time per week)^c,d^				
Yes	58 (93.5)	174 (98.3)	55 (94.8)	166 (98.8)
No	4 (6.5)	3 (1.7)	3 (5.2)	2 (1.2)
Previous mental health clinical diagnosis or treatment^c,d^				
Yes	26 (44.1)*	37 (22.4)	29 (49.2)*	36 (21.6)
No	33 (55.9)	128 (77.6)	30 (50.8)	131 (78.4)
Ever visited campus psychologist^d^				
Yes	30 (50.8)	69 (41.3)	23(46.9)	56 (44.1)
No	29 (49.2)	98 (58.7)	26 (53.1)	71 (55.9)
Perceived stigma of mental health issues at college^d^				
Strongly agree/Agree	15 (26.3)	109 (68.1)	23 (41.1)*	43 (26.7)
Disagree/Strongly disagree	42 (73.7)	51 (31.9)	33 (58.9)	118 (73.3)
Perceived stress at current college higher than other universities				
Yes	18 (31.0)	50 (30.3)	19 (32.2)	52 (31.0)
No	40 (69.0)	115 (69.7)	40 (67.8)	116 (69.0)

*p-value <0.05.

*Notes.*^a^May not sum to N because some participants did not complete this measure.

^b^Not included in predictive model due to collinearity.

^c^Included in predictive model for CES-D.

^d^Included in predictive model for DASS-21.

The absence of a self-reported strong social support group (adjusted odds ratio [AOR] 4.3, 95% CI 1.4—13.7) and previous history of a mental health disorder (AOR 4.8, 95% CI 1.9—12.4) remained significant in the backward and stepwise elimination models for current depression by CES-D (Table 3). The absence of a self-reported strong social support group (AOR 5.8, 95% CI 1.3—25.7) and previous history of a mental health disorder (AOR 5.8, 95% CI 1.9—17.7) also remained significant in the DASS-21 backward and stepwise elimination models. In addition, self-reported employment (AOR 4.8, 95% CI 1.4—15.5) and overall health (AOR 5.6, 95% CI 1.1—29.4) remained significantly associated with DASS-21 depression.

**Table 3:** Multivariable analysis for predictors of CES-D/DASS-21 depression

	**Measure of depression**			
	**CES-D**		**DASS-21**	
**Participant characteristic**	**OR (95% CI)**	**AOR**^**a**^**(95% CI)**	**OR (95% CI)**	**AOR**^**a**^**(95% CI)**
Employment				
Yes	--	--	1	1
No			2.1 (1.0, 4.4)	4.8 (1.4, 15.5)
Self-reported overall health				
Excellent/Very good/Good	--	--	1	1
Fair/Poor			5.6 (2.0, 16.0)	5.6 (1.1, 29.4)
Strong social support group				
Yes	1	1	1	1
No	4.5 (1.7, 12.1)	4.3 (1.4, 13.7)	7.1 (2.5, 19.8)	5.8 (1.3, 25.7)
Previous mental health clinical diagnosis or treatment				
Yes	3.8 (1.8, 8.0)	4.8 (1.9, 12.4)	4.9 (2.3, 10.3)	5.8 (1.9, 17.7)
No	1	1	1	1

*Note.*^a^Adjusted models were derived from backward and stepwise elimination techniques performed in SAS version 9.3 (SAS Institute, Cary, NC). Covariates entered into the elimination algorithm are indicated in Table 2.

In separate non-elimination multivariable models, poor sleep quality was significantly associated with both CES-D and DASS-21 measures of depression after adjusting for confounders (Table 4). Students who reported poor or extremely poor sleep quality were more likely to also report prevalent CES-D scores ≥16 (AOR 2.8, 95% CI 1.3--5.8). Similarly, the odds of prevalent DASS-21 depression symptoms among students with poor or extremely poor sleep quality was 2.8 (95% CI 1.4--5.8) times that of students who self-reported sleep to be excellent, good, or okay.

**Table 4:** Depression symptoms and selected study participant characteristics: Multivariable results for adjusted prevalence odds of depression

	**Measure of depression**			
	**CES-D**		**DASS-21**	
**Participant Characteristic**	**OR (95% CI)**	**AOR**^**a**^**(95% CI)**	**OR (95% CI)**	**AOR**^**a**^**(95% CI)**
Sleep quality				
Excellent/Good/Okay	1	1	1	1
Poor/Extremely poor	3.3 (1.7, 6.3)	2.8 (1.3, 5.8)	3.4 (1.8, 6.6)	2.8 (1.4, 5.8)
Residence				
On-campus	1	1	1	1
Off-campus	1.7 (0.9, 3.5)	1.9 (0.8, 4.7)	1.3 (0.6, 2.7)	1.3 (0.5, 3.3)
Self-reported overall health				
Excellent/Very good/Good	1	1	1	1
Fair/Poor	6.3 (2.6, 15.1)	3.9 (1.5, 10.7)	4.2 (1.8, 10.1)	2.7 (1.0, 7.2)

*Note.*^a^Multivariable adjusted models included all variables reported in the table and age. Adjustment was based on previous literature and from directed acyclic graphs.

## Discussion

In our cross-sectional survey, 26% of undergraduate students attending a women’s college self-reported prevalent depression by both CES-D (n = 240) and DASS-21 (n = 227) instruments. Previous history of a mental disorder and the absence of a self-reported strong social support group remained significant predictors of depression in backward and stepwise elimination models for depression by both instruments. In adjusted causal models, students with poor sleep quality had approximately 2.8 times the odds of depression (by both CES-D and DASS-21) compared to that of students who reported higher quality sleep.

Previous studies among female US college students have reported the prevalence of current depression to be between 16.6% and 41.3% [21,33,34], indicating our results from a women’s college are within the range of results from other co-ed institutions. Regestein et al. [12] and Herman et al. [34] reported mean CES-D scores from surveys of female college students of 17.9 (SD 10.9) and 15.4 (SD 9.7), respectively, both higher than our observed mean CES-D score of 12.4 (SD 9.8). Previous studies have reported an association between depression and sleep quantity [35,36] and quality [35,37]. Similar to our findings of a strong relationship between sleep quality and depression, Regestein et al. [12] found that residential students attending a women’s college who self-reported trouble sleeping and sleepiness were significantly associated with higher CES-D scores (p-values < 0.01). Poor sleep quality and depression have been found to have a bidirectional relationship among youth and young adults [18]. Studies correlating depression and sleep latency and interruptions suggest that specific aspects of sleep quality may be related to depressive symptoms [35,37]. Our finding that the absence of a strong social support group is associated with depressive symptoms is also consistent with previous studies that found more CES-D defined depression among both adults and college students with lower self-perceived emotional support from family and social networks [38-40].

### Limitations

This study is subject to limitations. The cross-sectional study design provides inference only to prevalent associations, without establishing temporality, between student characteristics and depressed mood. Misclassification of depression was possible because symptoms were self-reported; nonetheless, we used two extensively validated instruments to measure depression and the anonymous nature of the survey likely reduced the potential for this bias. Both CES-D and DASS-21 instruments assess short-term depressive mood, consequently the outcome may not reflect long-term associations that would result in clinical depression. Because the study population included undergraduate students attending a women’s college, the generalizability of the results to co-ed colleges may be limited. In addition, because the survey was conducted at only one undergraduate women’s college and the response rate was 37%, the generalizability of our findings to other female-only institutions of higher education may be limited.

## Conclusion

In large 2012 national survey of colleges, 47.6% of undergraduate students reported that they had not received information regarding depression or anxiety from their academic institution, while 49.0% reported interest in receiving this information [1]. Given the increasing prevalence of depressive symptoms among college students, systematic screening for mental health concerns at college health centers should be prioritized as a public health issue [20]. Systematic screening that incorporates measures of sleep quality and quantity may improve detection of depression and other mental health disorders [41]. Further, sleep interventions may help control and prevent symptoms of these disorders [42].

Our findings suggest that estimates of current depression prevalence among female US students attending a women’s college are similar to those of current depression prevalence among female US coeducational college students. Our findings support the importance of providing undergraduate mental health resources and the need for improved knowledge of the relationships between social support, sleep quality, and depression.

## Competing interests

The authors indicate that they have no financial or other competing interests to declare.

## Authors' contributions

KTW performed statistical analyses and drafted the tables. AEB, AA, DYD, and DMJ performed descriptive statistics. MJM led data collection and analyses. All authors contributed to conception and design of the study, data collection, and drafting the manuscript. All authors read and approved the final manuscript.

## Pre-publication history

The pre-publication history for this paper can be accessed here:

http://www.biomedcentral.com/1472-6874/14/8/prepub
